# Cutting-Edge Imaging of Cardiac Metastases from Neuroendocrine Tumors: Lesson from a Case Series

**DOI:** 10.3390/diagnostics12051182

**Published:** 2022-05-09

**Authors:** Soraya El Ghannudi, Eric Ouvrard, Nidaa Mikail, Benjamin Leroy Freschini, Thomas H. Schindler, Alessio Imperiale

**Affiliations:** 1 Nuclear Medicine, Institut de Cancérologie de Strasbourg Europe (ICANS), University Hospitals of Strasbourg, 67093 Strasbourg, France; e.ouvrard@icans.eu (E.O.); bleroyfreschini@gmail.com (B.L.F.); 2 Department of Radiology, University Hospitals of Strasbourg, 67098 Strasbourg, France; 3 Nuclear Medicine, ENETS Centre of Excellence, Beaujon Hospital (APHP), 92110 Clichy, France; m.nidaa@yahoo.fr; 4 Division of Nuclear Medicine, Mallinckrodt Institute of Radiology, Washington University, St. Louis, MO 63110, USA; thschindler@wustl.edu; 5 Molecular Imaging—DRHIM, IPHC, UMR 7178, CNRS/Unistra, 67093 Strasbourg, France

**Keywords:** neuroendocrine, cardiac metastasis, positron emission tomography, ^68^Ga-DOTATOC, ^18^F-DOPA, cardiac magnetic resonance, echocardiography, cardiac imaging

## Abstract

With the increasing availability of high-performance medical imaging for the management of patients with neuroendocrine tumors (NETs), a progressive growth of asymptomatic and incidentally detected cardiac metastases (CMs) has been observed in the recent years. In clinical practice, CMs of NENs are often incidentally detected by whole-body ^68^Ga-labeled somatostatin analogs or ^18^F-fluorodihydroxyphenylalanine positron emission tomography/computed tomography, and afterwards accurately characterized by cardiac magnetic resonance (CMR) and/or gated cardiac computed tomography when CMR is contraindicated or not available. The interpreting physician should familiarize with the main imaging features of CM, a finding that may be encountered in NETs patients more than previously thought. Herein, we present a case series of four patients with CMs from small-intestine NETs highlighting strengths and weaknesses of a multimodality imaging approach in clinical practice.

## 1. Introduction

Neuroendocrine tumors (NETs) are heterogeneous epithelial tumors with neuroendocrine differentiation, most frequently originating from the gastrointestinal tract, pancreas, and lung. Within the gastrointestinal tract, the small intestine represents the first site of primary tumor origin [1]. On first presentation, most patients have distant metastases, generally in the liver. The increased availability of highly sensitive molecular imaging modalities allowed the detection of uncommon metastatic sites (including heart), with new concerns about patient therapeutic management and prognosis [2].

Cardiac metastases (CMs) from NENs generally appear late in the course of the disease. Nowadays, the knowledge of CMs prevalence, clinical characteristics, diagnostic strategy, treatment modalities, and related response to treatment is still limited. Their incidence is estimated to be 1–4%, but is likely higher because CMs are often asymptomatic and do not undergo specific work-up for cardiac lesion research [3]. Most CMs originate from NETs of the gastrointestinal tract and have been described most often in patients with well-differentiated functioning small-intestine neuroendocrine tumors (si-NETs). The need for a careful echocardiographic monitoring during follow-up to detect carcinoid heart disease, a possible complication of functioning si-NETs [4], may contribute to understand this association. However, no relationship between cardiac metastasis and carcinoid valve disease has been documented [5].

CMs from si-NETs appear as non-infiltrative and homogenous masses involving the right ventricle, the left ventricle, and the inter-ventricular septum in about 40%, 53%, and 7% of cases, respectively [6]. CMs have heterogeneous clinical presentation ranging from asymptomatic incidental discovery to life-threatening symptoms, such as right heart failure and acute pulmonary edema [7]. CMs can be also responsible for atrial fibrillation, ventricular tachycardia, and ventricular fibrillation; therefore, in case of sudden onset of arrhythmia or heart failure in a patient with cancer, the presence of CMs must be ascertained. Rarely, pericardial effusion or cardiac tamponade could be the early clinical presentations of a cardiac involvement of malignant disease, although 90% of lesions are clinically silent [7].

Positron emission tomography/computed tomography (PET/CT) represents a front-line imaging examination in NENs patients. In clinical practice, CMs of NENs are often incidentally detected by whole-body ^68^Ga-labeled somatostatin analogs (^68^Ga-SSAs) or ^18^F-fluorodihydroxyphenylalanine (^18^F-DOPA) PET/CT, and afterwards accurately characterized by cardiac magnetic resonance imaging (CMR) and/or gated cardiac computed tomography (Cardiac-CT) when CMR is contraindicated or not available. The interpreting physician should familiarize with the main imaging features of CM, a finding that may be encountered in NET patients more than previously thought. Herein, we present a case series of four patients with CMs from small-intestine NENs (si-NETs) highlighting strengths and weaknesses of a multimodality imaging in clinical practice.

## 2. CASES Presentation

### 2.1. Patient 1

**Figure 1:**
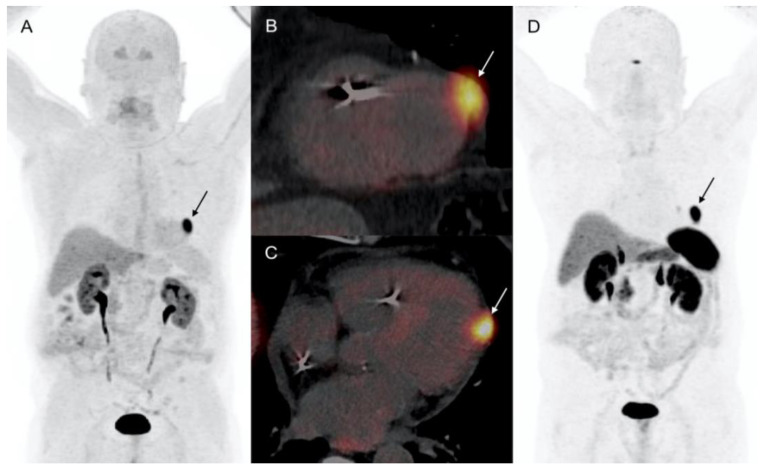
Patient 1. 72-year-old patient with metastatic si-NET. LV CM (arrows) showed intense uptake of both ^18^F-DOPA and ^68^Ga-DOTATOC. (**A**) ^18^F-DOPA PET MIP (anterior projection). (**B**,**C**) ^18^F-DOPA PET/CT short axis, and 4 chamber view. (**D**) ^68^Ga-DOTATOC PET MIP (anterior projection).

**Figure 2:**
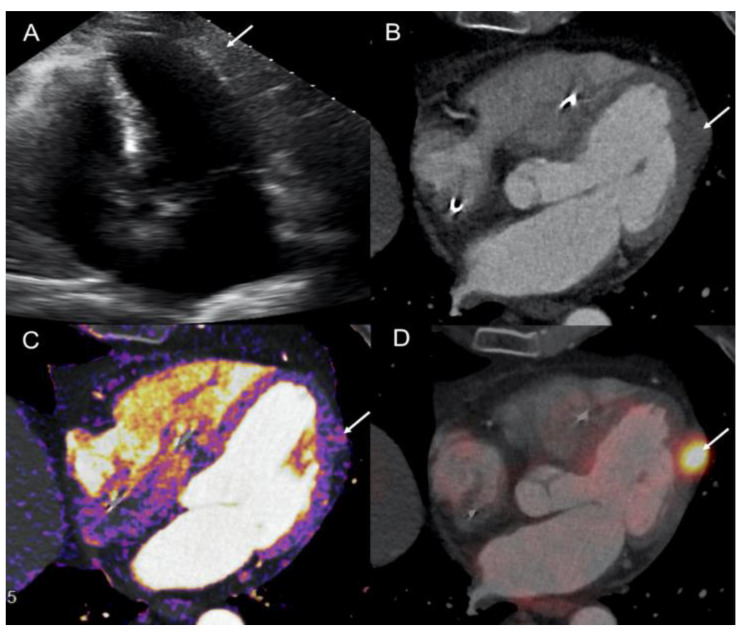
Patient 1. TTE (5-chambers view) showed isoechogeneous mass (arrow) in the latero-apical LV wall (**A**). Contrast-enhanced ECG-gated cardiac-CT (4-chambres view) confirmed the presence of a myocardial metastasis (arrow) with pericardial infiltration (**B**). No significant tumoral contrast-enhancement was detectable at visual analysis. Conversely, iodine mapping analysis revealed a heterogeneous, mild, and peripheral enhancement of ventricular metastasis (arrow) (**C**). Fused ^18^F-DOPA PET/Contrast-enhanced ECG-gated cardiac-CT images (**D**, 4-chamber view).

A 72-year-old male with arterial hypertension referred to our Institution for further evaluation of chronic abdominal pain. The patient had previous history of atrial fibrillation complicated with ischemic cerebral disease, and a third-degree atrioventricular block requiring a pacemaker implantation. Three-phase, contrast-enhanced abdominal and pelvic computed tomography (CT) revealed multiple hepatic lesions and enlarged mesenteric lymph nodes. Laboratory evaluation showed an increased serum serotonin and chromogranin-A and urinary 5-hydroxyindoleacetic acid (5-HIAA) values. Due to suspicion of NET, the patient underwent ^111^In-labeled somatostatin analogs (pentetreotide) scintigraphy showing multiple foci of abdominal radiotracer uptake corresponding to mesenteric lymph nodes and hepatic metastases of segment VII. No primary tumor was visualized, thus ^18^F-DOPA PET/CT was performed, confirming the lymphatic and hepatic metastatic spread, and revealing a pathologic focal uptake in terminal ileum (Figure 1). Moreover, an intense focal uptake of ^18^F-DOPA (SUVmax: 18.9) was showed in the lateral wall of the left ventricle (LV) suggesting a cardiac metastasis. The ECG was unremarkable, and transthoracic cardiac echocardiography (TTE) performed immediately after PET/CT and 3 months later revealed no LV lesions. The patient had neither chest pain nor dyspnea, left ventricular regional kinetics and ejection fraction were preserved (60%), and no signs of carcinoid heart disease were objectified. The patient was surgically treated and the pathology report confirmed the presence of si-NET (grade 1, Ki67: 1–2%) with nodal and liver metastases. Treatment with cold somatostatin analogs was started (lanreotide 90 mg, every 4 weeks). Both ^18^F-DOPA and ^68^Ga-DOTATOC PET/CT (SUVmax: 37.1) performed during follow-up showed the persistence of LV focal uptake (Figure 1). CMR was contraindicated due to the non-MRI-compatible pacemaker. Thus, a contrast ECG-gated cardiac CT was performed confirming the presence of a myocardial metastasis of 22-mm of the LV latero-apical wall with pericardial infiltration without effusion (Figure 2). Quantitative dual-energy CT iodine mapping was performed to characterize LV lesion, presenting mild, heterogenous, and peripheral iodine contrast enhancement. In light of the results obtained, previous TTE images were retrospectively analyzed, and LV lesion was recognized. The patient was asymptomatic and without cardiac hemodynamic consequences, therefore, a non-surgical attitude was preferred in this case. CM remained stable and asymptomatic during 36-months follow-up.

### 2.2. Patient 2

**Figure 3:**
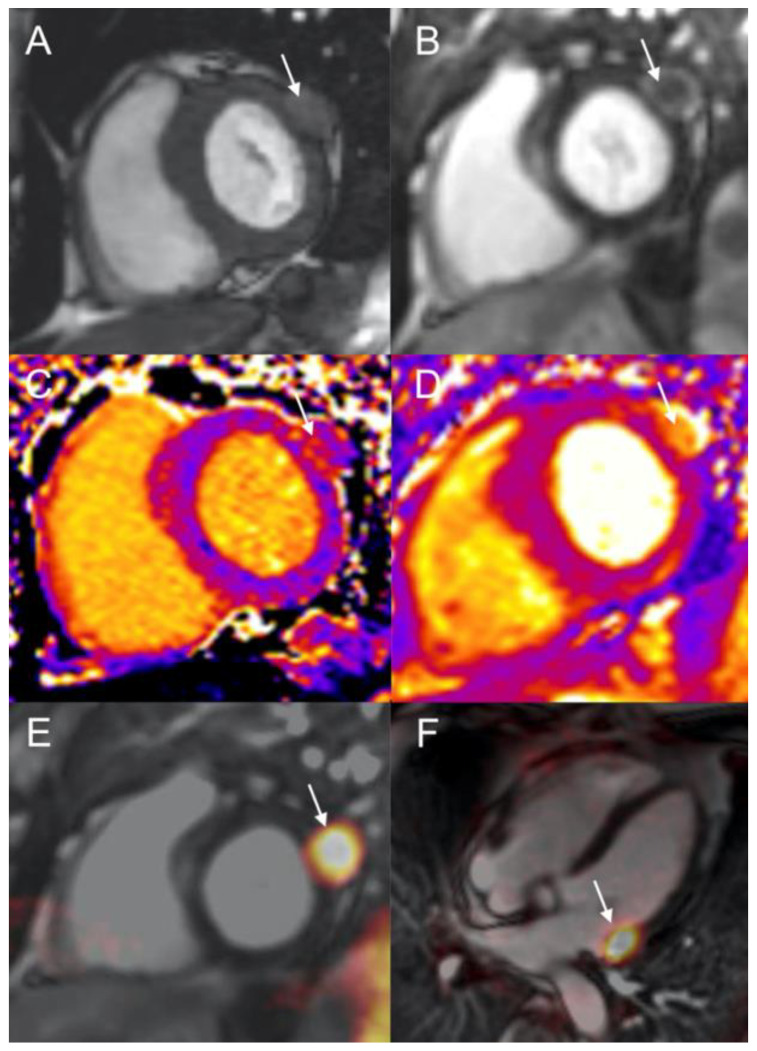
Patient 2. CMR showing a NEN metastasis of lateral basal LV wall (arrows) appearing as 25-mm nodule with intermediate intensity on SSFP T1-weighted images (**A**), with peripheral contrast-enhancement on phase sensitive inversion recovery sequence (PSIR) (**B**). T1- (**C**) and T2-mapping images (**D**) revealed an increased T1 (1172 ms), and T2 (81 ms). Fused ^68^Ga-DOTATOC PET/CMR: short axis (**E**) and 4-chamber view (**F**).

**Figure 4:**
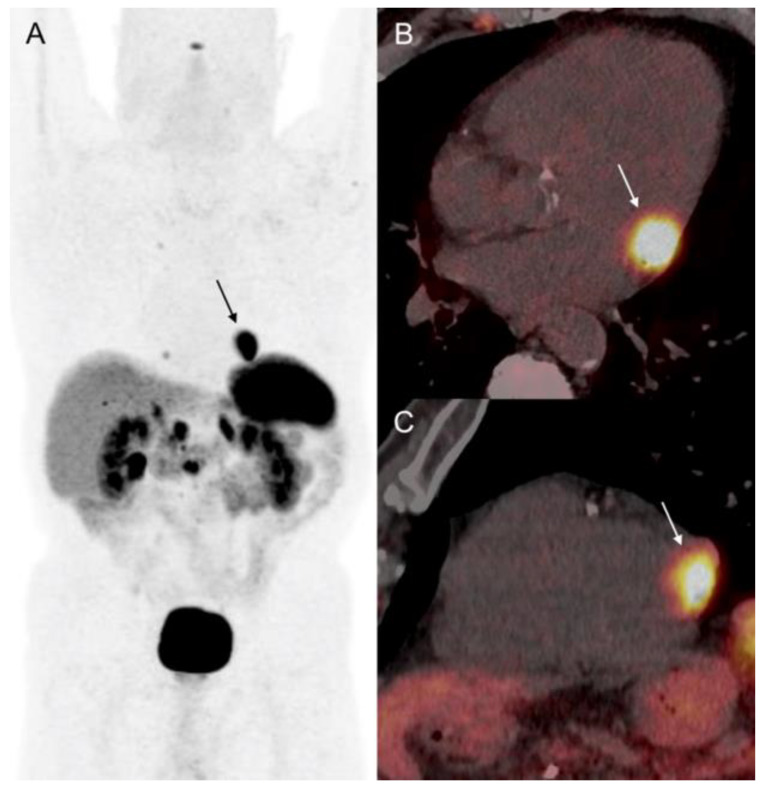
Patient 2. 74-year-old patient with metastatic si-NET and LV CM (arrows) showing intense uptake of ^68^Ga-DOTATOC ((**A**) MIP anterior projection, (**B**) PET/CT horizontal long axis, and (**C**) PET/CT short axis).

A 74-year-old man with metastatic si-NET was addressed to our institution for further investigations during post-surgical follow-up. The patient’s story began 2 years earlier with abdominal pain and the discovery of a mesenteric mass and a unique liver lesion on a CT scan. Histology of the liver nodule revealed a metastasis from a well-differentiated NET. The patient had no clinical signs of serotonin hypersecretion (carcinoid syndrome) and biological analysis showed isolated mild increase of chromogranin-A. ^111^In-pentetreotide scintigraphy showed a pathological uptake in known liver and mesenteric metastases, and revealed a focal radiotracer uptake in the lateral wall of LV. The patient was asymptomatic, ECG was normal, and routine TTE showed no nodular ventricular abnormalities. LV ejection fraction was normal (70%). The patient was surgically treated and pathological analysis revealed a grade 1 (ki67: 1%) unifocal si-NET with metastatic lymph-nodes, a mesenteric mass, and one liver metastasis. Unfortunately, synchronous colic adenocarcinoma and intrahepatic cholangiocarcinoma were also detected, the latter requiring capecitabine chemotherapy. No adjuvant therapy was decided for the si-NET. Follow-up ^111^In-pentetreotide scintigraphy performed about one year later, confirmed the persistence of LV focal intense uptake without other abnormalities. CMR was, thus, performed to confirm the presence of a CM (Figure 3). Steady-state free precession sequences revealed a tissular nodule of 25 mm originating from lateral basal LV wall. There was no LV wall motion abnormality, and end-diastolic volume was normal (67 mL/m^2^, N < 95 mL/m^2^). Cardiac lesion presented intermediate hyper-intensity on T2 weighted fat-saturated dark-blood images. The T1- and T2-mapping analysis revealed long T1 and T2 (1172 ms, 81 ms respectively) values, and moderate increase of extracellular volume (31%, N < 27%). On post-gadolinium contrast inversion recovery sequences, LV tumor presented with low, heterogeneous, and mainly peripheral contrast-enhancement when compared to normal myocardium. LV metastasis also showed a high focal uptake of ^68^Ga-DOTATOC (SUVmax: 25.6) in the lateral-basal segment of LV wall (Figure 4) in concordance with ^111^In-pentetreotide scintigraphy and CMR findings. The patient started treatment with cold somatostatin analogues (lanreotide 90 mg, every 4 weeks). Cardiac lesion remained stable and patient asymptomatic during 36 months of follow-up.

### 2.3. Patient 3

**Figure 5:**
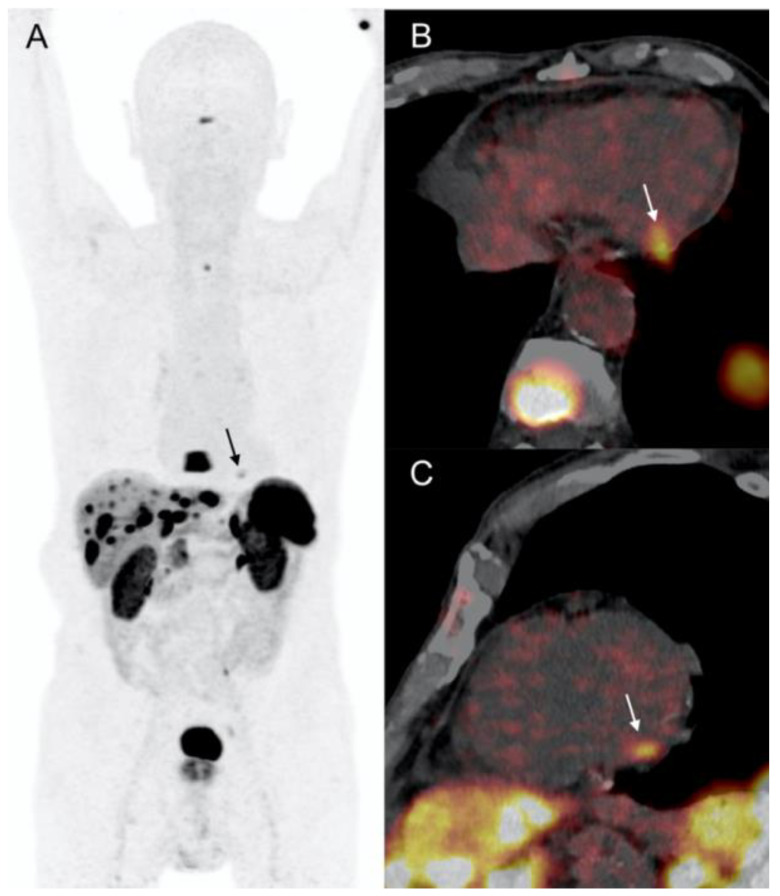
Patient 3. 75-year-old patient with metastatic si-NET and LV CM (arrow). Cardiac lesion showed pathological uptake of ^68^Ga-DOTATOC ((**A**) MIP anterior projection, (**B**) PET/CT horizontal long axis, (**C**) PET/CT short axis).

A 75-year-old man with history of metastatic si-NET (grade 2, ki67: 4%) treated about 15 years earlier by right ileo-colectomy, liver metastasectomy, and chemoembolization, referred for routine follow-up evaluation during cold somatostatin analogs treatment (lanreotide 90 mg) on a monthly basis. The patient had chronic atrial fibrillation, hypertension, dyslipidemy, diabetes mellitus, moderate obesity, chronic kidney disease, and one vessel coronary artery disease treated by stenting of the left anterior descending coronary with effective long-term results. Biology showed elevated values of serum serotonin and chromogranin-A, and a moderate increase of 5-HIAA. Contrast-enhanced CT of chest, abdomen and pelvis confirmed the presence of hepatic and nodal metastases, globally stable. ^68^Ga-DOTATOC PET/CT revealed several bilobar liver metastases and unique dorsal vertebral localization (Figure 5).

**Figure 6:**
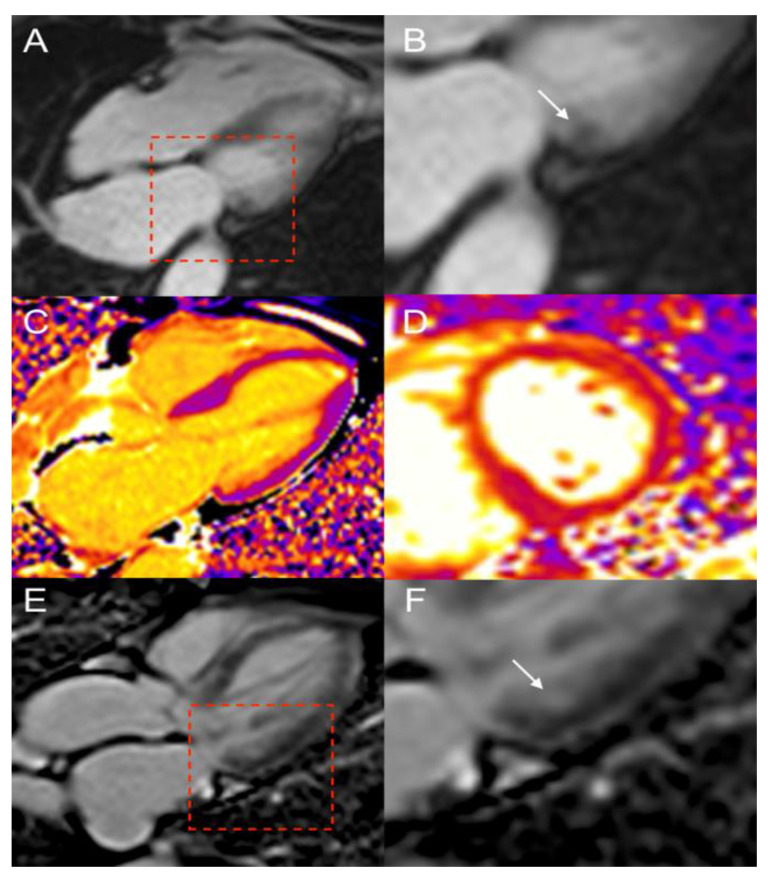
Patient 3. CMR showing a few millimeters nodule imbedded in infero-lateral basal LV wall (arrow). Steady-state free precession sequences (**A**,**B**). T1- and T2-mapping analysis failed to detect the LV wall metastasis mainly due to the small lesion size (**C**,**D**). CM presented intense gadolinium enhancement on delayed post-contrast inversion recovery sequences (**E**,**F**).

Moreover, pathological focal ^68^Ga-DOTATOC uptake (SUVmax: 5.6) was identified in the basal segment of the inferolateral wall of the LV. ECG showed QS aspect on anterior leads and atrial fibrillation. TTE failed to detect any LV wall lesion, and revealed a normal LV function. The patient underwent CMR showing a 6-mm nodule in the inferior-lateral wall on steady-state free precession (SSFP) sequences, with intense enhancement on post-gadolinium contrast inversion recovery images (Figure 6). T1- and T2-weighted sequences, and T1- and T2-mapping analysis showed no LV abnormalities. There was no late gadolinium enhancement with ischemic topography. There were no LV walls motion abnormalities, and both LV ejection fraction and end-diastolic volume were normal (LVEF: 69%; EDLVV: 70 mL/m^2^, N < 95 mL/m^2^). Thus, the diagnosis of incidental CM of well-differentiated NEN was retained. Systemic metastatic disease remained stable under lanreotide on follow-up ^68^Ga-DOTATOC PET/CT. CM was asymptomatic and cardiac function was preserved during 12-months follow-up.

### 2.4. Patient 4

**Figure 7:**
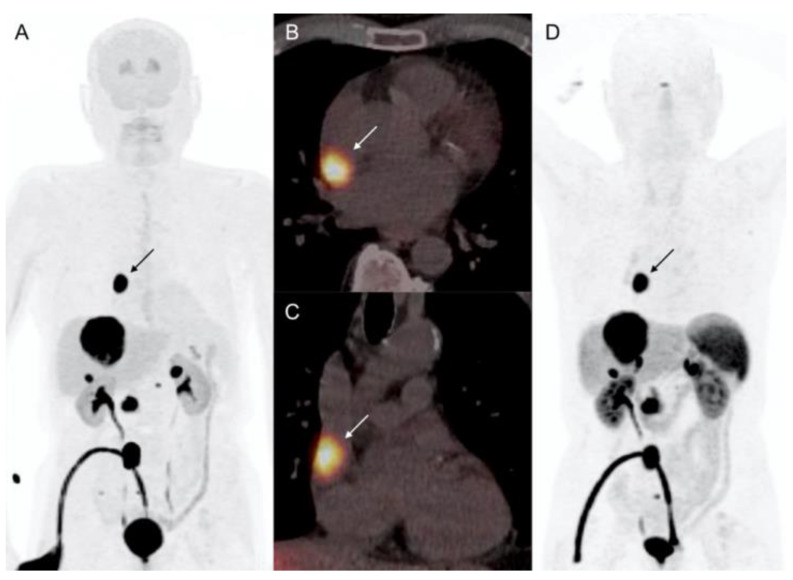
Patient 4. 76-year-old patient with metastatic si-NET. LV CM (arrows) showed intense uptake of both ^18^F-DOPA and ^68^Ga-DOTATOC. (**A**) ^18^F-FDOPA PET MIP (anterior projection). (**B**,**C**) ^18^F-FDOPA PET/CT short axis, and 4 chamber view. (**D**) ^68^Ga-DOTATOC PET MIP (anterior projection).

**Figure 8:**
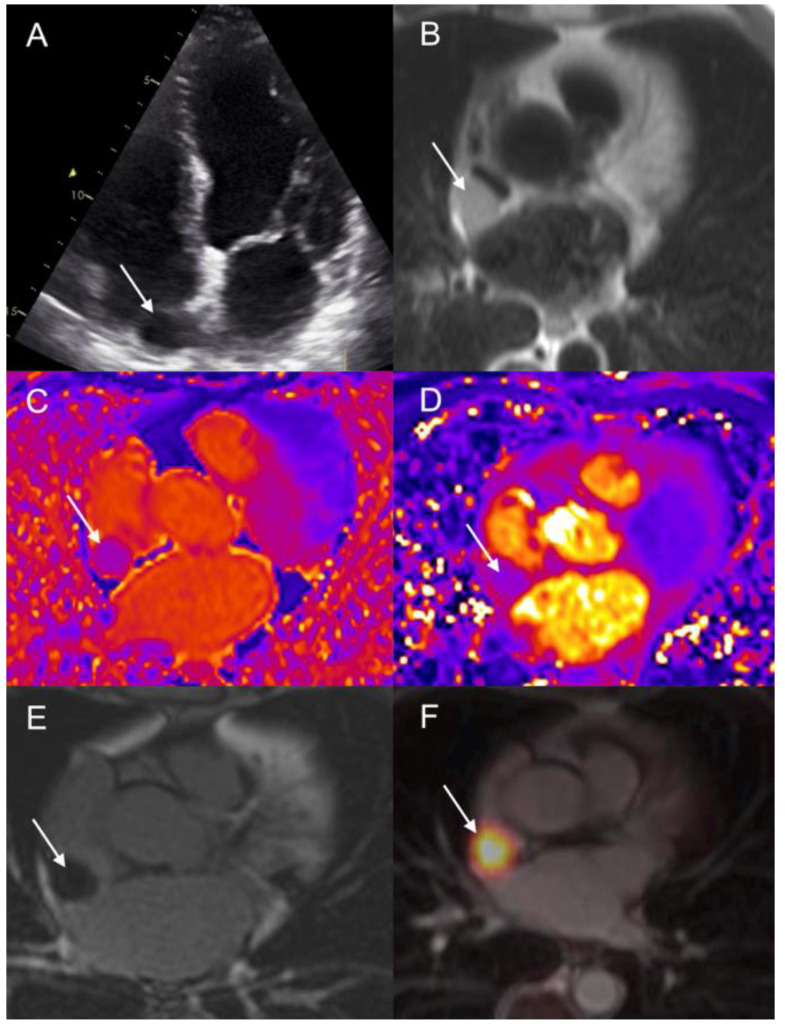
Patient 4. TTE (4-chamber view) showing hypoechogeneous lesion on the right side of inter-atrial septum ((**A**) arrow). Lesion showed intermediate signal intensity on T1-weighted dark-blood CMR images ((**B**) arrow). T1- (**C**) and T2-mapping (**D**) analysis revealed short T1 and moderately increased T2 values (**D**). CM showed a slight peripheral enhancement on delayed post-contrast inversion recovery sequences (**E**). Fused ^68^Ga-DOTATOC PET/CMR images (axial slice) (**F**).

A 76-year-old man with a 20-year history of right ileo-colectomy for metastatic si-NET (grade 2, ki67: 5%) referred for routine evaluation. The patient presented with elevated values of urinary 5-HIAA and a carcinoid syndrome treated by cold somatostatin analogs (lanreotide 120 mg) on a monthly basis. ^18^F-DOPA PET/CT revealed multiple liver, nodal, and bone metastases (Figure 7). A focally increased ^18^F-DOPA accumulation (SUVmax: 44.8) was also detected in the interatrial septum suggesting metastatic localization. The patient was asymptomatic without chest pain or sign of cardiac insufficiency. ECG demonstrated sinus rhythm at 72 beats per minutes, normal axis and PQ interval, normal R/S transition, and repolarization. TTE ruled-in a tiny thickening of posterior interatrial septum (16 mm) (Figure 8), corresponding to the ^18^F-DOPA uptake. LV function was preserved without sign of carcinoid heart disease. Following CMR confirmed the presence of a tissular nodule of 22-mm in the right side of the inter-atrial septum with intermediate hyper-intensity on T1 weighted sequence (Figure 8). T1- and T2-mapping analysis revealed short T1 (854 ms, normal myocardium: 1000) and moderate increase of T2 myocardial relaxation times (64 ms, normal myocardium: 50 ms). There were no LV walls motion abnormalities, and both LVEF and LV volume were normal (LVEF: 69%; EDLVV: 76 mL/m^2^, N < 95 mL/m^2^). On post-gadolinium contrast inversion recovery sequences (PSIR), the tissular nodule presented mild peripheral contrast enhancement. During follow-up, metastatic disease progressed under lanreotide on serial CT scans and patient was scheduled for PRRT. CM was easily detected by ^68^Ga-DOTATOC PET/CT (Figure 7) performed before radiometabolic treatment (SUVmax: 34.1). CM remained asymptomatic during follow-up and no deterioration of cardiac function was observed.

## 3. Discussion

In the evaluation of patients with metastatic NETs, a multidisciplinary strategy is usually used, taking into account the clinical context, both strengths and limitations of each diagnostic modality, and the local availability. Radiological investigations provide detailed anatomical information that is indispensable for primary tumor detection, metastases identification, and surgical planning optimization. Nuclear medicine techniques allow non-invasive characterization of tumoral functional status and variability at the molecular and cellular level from the analysis of uptake intensity and kinetics of target-specific radiotracers. Molecular imaging techniques are also very sensitive and can detect disease at an early stage. Nowadays, anatomic and functional imaging are usually combined into «hybrid» modalities, such as PET/CT. Positron Emission Tomography/Magnetic Resonance Imaging (PET/MRI) is also available and may be effective, particularly in patients with cardiac metastases although its availability in clinical routine is still limited [8].

In NETs, the overexpression of transmembrane somatostatin receptors (SSTR), as well as the cellular ability to take-up amine precursors, have encouraged the development of specific radiotracer for PET/CT investigations, such as ^68^Ga-labeled somatostatin analogs (comprehensively called ^68^Ga-DOTA-peptides) and ^18^F-DOPA. In the recent years, ^68^Ga-DOTA-peptides and ^18^F-DOPA whole-body PET/CT emerged as the current gold standard for NET patients, currently performed at primary staging and during follow-up [9]. PET with somatostatin analogs have almost replaced gamma camera techniques, and ^68^Ga-DOTA-peptides PET/CT has proven to be successful in the evaluation of well-differentiated NETs. Moreover, it is also strongly connected to the use of targeted peptide radionuclide therapy (PRRT) in inoperable and progressive metastatic patients. ^18^F-DOPA PET/CT has excellent sensitivity for low-grade serotonin-secreting si-NETs that are primarily related to increased tumor biosynthesis of serotonin [10]. Thus, ^18^F-DOPA PET/CT provides a specific molecular signature related to serotonin secretion. In a clinical routine, most CMs from NETs are detected incidentally during routine PET/CT investigations and subsequently characterized by focused morphologic imaging to accurately define tumor localization, size, and its anatomic situation. Recently, a progressive growth of cardiac lesions identification has been observed, especially in multimetastatic patients [11,12,13]. In a recent retrospective study, the prevalence of CM detected on ^18^F-DOPA PET/CT has been reported to be 13% [11].

Transthoracic echocardiography (TTE) is widely used due to its high availability and lack of patient exposure to ionizing radiation. However, TTE seems unreliable for the initial detection of primary or metastatic cardiac involvement mainly because of its inadequate spatial resolution and its operator-dependent nature making it highly subjective to interpretive error. TTE remains clinically useful for determining cardiac function and eventual valvular involvement, particularly in patients with functioning si-NET and carcinoid syndrome [6].

CMR is one of the most comprehensive imaging modalities for the diagnosis and characterization of cardiac masses [14,15]. It allows for high spatial resolution and multi-planar imaging providing accurate anatomic location, mass mobility, and tissue characterization using gadolinium perfusion images, T1, and T2 mapping sequences. CMR is useful for risk stratification and clinical decision making for patients with cardiac masses [16]. It has been reported that the accuracy of CMR in differentiating between neoplastic and non-neoplastic mass, and between benign and malignant tumor was 100% and 96%, respectively, with pathological validation [16]. CMs show variable enhancement profile after contrast-media administration [16,17]. Larger masses commonly present heterogeneous enhancement mainly due to tumor necrosis secondary to tissue hypoxia [18].

ECG-gated cardiac CT (cardiac-CT) is a reliable complementary tool to CMR in the evaluation of cardiac masses [19]. Although CMR remains the first-choice imaging modality due to its superior tissue characterization and non-irradiating nature, cardiac-CT offers a valuable diagnostic alternative, superior to echocardiography, in those patients with contraindications to CMR. In addition, CT better depicts calcification and has a higher spatial resolution than CMR, allowing better definition of the margins of the mass and its anatomic relationship to adjacent normal structures, which is of particular importance for preoperative strategy planning [20].

To date, no standard treatment has been defined for CMs from NENs. Specific treatment for CMs is not needed in most cases due to the lack of functional cardiac involvement. Typically, focused treatment is considered in patients with clinical symptomatology or in those cases with impaired myocardial function due to valvular obstruction or intracavitary metastasis with negative hemodynamic effects. On the other hand, patients with CMs present with advanced metastatic disease and need for a systemic treatment, such as targeted molecular therapy, cold somatostatin analogs (SSAs), or peptide radionuclide therapy (PRRT) [1,2]. In this respect, sufficient levels of SSTR expression have to be documented before peptide receptor radionuclide therapy (PRRT). Thus, theragnostic imaging of SSTR is a prominent indication for ^68^Ga-DOTA-peptide PET/CT in patients with metastatic NETs allowing a robust patient selection before PRRT.

The prognostic implication of cardiac involvement in patients with NETs is still unknown. The clinical significance of cardiac metastases incidentally revealed during imaging investigations may not be of high significance, due to the relatively slow evolution of the disease, leading to a comparable survival in NET patients without cardiac metastatic spread. In general, the early NET diagnosis and confirmation of cardiac metastases would allow the introduction of different treatments, in the earliest stages of the disease reducing the impact on the heart with potential better therapeutic outcome. Although evidence suggesting that the presence of CMs does not negatively impact the overall survival, the real consequence of CMs should be evaluated for each patient taking into account metastatic localization, lesion size as well as the related clinical symptomatology [5,12].

We acknowledge that the lack of definitive cardiac histological standard for imaging comparator could represent a limitation of our work. Based on recommendations [21], the endomyocardial biopsy is reasonable in the setting of suspected cardiac tumors (Class of Recommendation IIa, Level of Evidence C) if: (1) the diagnosis cannot be established by noninvasive imaging modalities or less invasive (non-cardiac) biopsy; (2) tissue diagnosis can be expected to influence the course of therapy; (3) the chances of successful biopsy are believed to be reasonably high; and (4) the procedure is performed by an experienced operator. In our patients, there were no cardiovascular clinical symptoms. Indeed, the cardio-vascular physical examination, electrocardiogram, and left ventricular ejection fraction (LVEF) were normal. There was no previous history of cardiovascular disease or inflammatory disease. Moreover, NET diagnosis was previously performed by pathological examination of non-cardiac tissue specimen (often liver metastasis) obtained after biopsy. Hence, the indication for myocardial biopsy or cardiac surgery was not retained and the non-invasive diagnosis of CM was conducted according to the existent clinical recommendations [21], mainly using specific PET radiotracers as ^18^F-DOPA and ^68^Ga-DOTA-peptides targeting the neuroendocrine phenotype [22]. According to a metanalysis [23], ^68^Ga-DOTA-peptides have high diagnostic accuracy in patients with NETs (sensitivity and specificity of of 93% and 85%, respectively). Moreover, ^18^F-DOPA-PET/CT yields good performances in the detection of NET. In a study including 61 patients with NETs, the sensitivity and specificity of ^18^F-DOPA PET/CT were 91% and 96%, respectively [24]. In a recent metanalyses focused on 112 patients with small intestine NET [25], the pooled sensitivity of ^18^F-DOPA PET/CT was 83%, 89%, and 95% on patient-based (PBA), region-based (RBA), and lesion-based analysis (LBA), respectively. ^68^Ga-DOTA peptide PET/CT showed sensitivity of 88%, 92%, and 82% on PBA, RBA and LBA, respectively. No significant differences were found between the two tracers on patient-based and region-based analysis. Thus, ^18^F-DOPA and ^68^Ga-DOTA-peptides PET/CT followed by multiparametric CMR for morphological assessment of the cardiac masses, left and right ventricle functional evaluation, and additional tissue characterization increased the confidence for a noninvasive diagnosis of asymptomatic CM in patients with multimetastatic NETs.

## 4. Conclusions

The following key points emerged from the analysis of the present series of NETs patients with CMs:

- CM is a possible asymptomatic complication of NETs, generally appearing late in the course of the disease, particularly in patients with well-differentiated functioning si-NETs, without relationship with carcinoid heart disease;
- ^68^Ga-DOTA-peptides and ^18^F-DOPA PET/CT are highly sensitive and specific for the initial diagnosis of CMs, which are usually detected incidentally during routine investigations;
- CMR and Cardiac-CT enable high-resolution, multiplanar imaging crucial for accurate anatomic localization, mass mobility assessment, and tissue characterization for noninvasive diagnosis confirmation;
- TTE remains clinically useful for cardiac function evaluation and tricuspid valve insufficiency detection (carcinoid heart disease), particularly in patients with functioning si-NETs and carcinoid syndrome;
- PET/MRI could represent a possible efficient “one-stop-shop” solution in patients with si-NETs.

## Data Availability

Not applicable.
